# An Anaerobic Environment Drives the Harboring of *Helicobacter pylori* within *Candida* Yeast Cells

**DOI:** 10.3390/biology11050738

**Published:** 2022-05-12

**Authors:** Kimberly Sánchez-Alonzo, Luciano Arellano-Arriagada, Humberto Bernasconi, Cristian Parra-Sepúlveda, Víctor L. Campos, Fabiola Silva-Mieres, Katia Sáez-Carrillo, Carlos T. Smith, Apolinaria García-Cancino

**Affiliations:** 1 Laboratory of Bacterial Pathogenicity, Department of Microbiology, Faculty of Biological Sciences, Universidad de Concepcion, Concepcion 4070386, Chile; kimsanchez@udec.cl (K.S.-A.); lucarellano@udec.cl (L.A.-A.); cparras@udec.cl (C.P.-S.); fabiolalsilva@udec.cl (F.S.-M.); csmith@udec.cl (C.T.S.); 2 Laboratorio Pasteur, Concepcion 4030635, Chile; hbernasconi@lpasteur.cl; 3 Laboratory of Environmental Microbiology, Department of Microbiology, Faculty of Biological Sciences, Universidad de Concepcion, Concepcion 4070386, Chile; vcampos@udec.cl; 4 Department of Statistics, Faculty of Physical and Mathematical Sciences, Universidad de Concepcion, Concepcion 4070386, Chile; ksaez@udec.cl

**Keywords:** *Helicobacter pylori*, oxygen, anaerobic condition, intracellular *H. pylori*, yeasts, *Candida*, environmental stress

## Abstract

**Simple Summary:**

*Helicobacter pylori* is a pathogen that is associated with a number of gastric pathologies and has adapted to the gastric environment. Outside this organ, stress factors such as oxygen concentration affect the viability of this bacterium. This study aimed to determine if changes in oxygen concentration promoted the entry of *H. pylori* into the interior of yeast cells of the *Candida* genus. Co-cultures of *H. pylori* and *Candida* strains in Brucella broth plus 5% fetal bovine serum were incubated under microaerobic, anaerobic, or aerobic conditions. Bacteria-like bodies (BLBs) were detected within yeast cells (Y-BLBs) by optical microscopy, identified by molecular techniques, and their viability evaluated by SYTO-9 fluorescence. Co-cultures incubated under the three conditions showed the presence of Y-BLBs, but the highest Y-BLB percentage was present in *H. pylori* J99 and *C. glabrata* co-cultures incubated under anaerobiosis. Molecular techniques were used to identify BLBs as *H. pylori* and SYTO-9 fluorescence confirmed that this bacterium remained viable within yeast cells. In conclusion, although without apparent stress conditions *H. pylori* harbors within *Candida* yeast cells, its harboring increases significantly under anaerobic conditions. This endosymbiotic relationship also depends mostly on the *H. pylori* strain used in the co-culture.

**Abstract:**

*Helicobacter pylori* protects itself from stressful environments by forming biofilms, changing its morphology, or invading eukaryotic cells, including yeast cells. There is little knowledge about the environmental factors that influence the endosymbiotic relationship between bacterium and yeasts. Here, we studied if oxygen availability stimulated the growth of *H. pylori* within *Candida* and if this was a bacterial- or yeast strain-dependent relationship. Four *H. pylori* strains and four *Candida* strains were co-cultured in Brucella broth plus 5% fetal bovine serum, and incubated under microaerobic, anaerobic, or aerobic conditions. Bacteria-like bodies (BLBs) within yeast cells (Y-BLBs) were detected by microscopy. *H. pylori* was identified by FISH and by PCR amplification of the *16S rRNA* gene of *H. pylori* from total DNA extracted from Y-BLBs from *H. pylori* and *Candida* co-cultures. BLBs viability was confirmed by SYTO-9 fluorescence. Higher Y-BLB percentages were obtained under anaerobic conditions and using *H. pylori* J99 and *C. glabrata* combinations. Thus, the *H. pylori*–*Candida* endosymbiotic relationship is strain dependent. The FISH and PCR results identified BLBs as intracellular *H. pylori*. Conclusion: Stressful conditions such as an anaerobic environment significantly increased *H. pylori* growth within yeast cells, where it remained viable, and the bacterium–yeast endosymbiotic relationship was bacterial strain dependent with a preference for *C. glabrata*.

## 1. Introduction

*Helicobacter pylori* is a Gram-negative, neutrophilic and microaerophilic bacterium with a helical morphology [1,2] and it infects above 50% of the population worldwide. An infection with this bacterium is mostly acquired during infancy and it is estimated that one third of the infant population is infected [3]

Differences in life conditions, such as hygiene levels, are probably responsible for the higher percentages of occurrences in developing countries [4,5]. This pathogenic microorganism colonizes the gastric epithelium, where it releases effector proteins, such as CagA and VacA, causing morphological changes in epithelial cells of the host and also stimulating the release of cytoplasmic components and interleukins, resulting in a strong inflammatory response which can lead to cell apoptosis with tissue damage [6,7]. The constant deterioration of the gastric epithelium, as a consequence of *H. pylori* infection, causes pathologies of the gastrointestinal tract, such as acute and chronic gastritis, peptic ulcer, gastric mucosa-associated lymphoid tissue (MALT) lymphoma, and gastric cancer [8,9], and the clinical manifestations are dependent on the immune condition of the host and on the virulence factors of *H. pylori* [3]. At present, there are multiple extra-gastric clinical manifestations attributed to infections by this pathogen, such as ischemic heart disease, anemia, insulin resistance, type 2 diabetes mellitus, and idiopathic thrombocytopenic purpura [9,10]. Thus, this pathogen is considered to have a high negative impact on human health, making it necessary to search for new treatment alternatives, such as probiotics or natural extracts (i.e., polyphenols) to confront the increase of *H. pylori* strains resistant to antibiotics [11,12,13,14,15,16]. However, despite its importance and the years of research dedicated to it, the dissemination routes and survival strategies used by this microorganism are still not completely understood [17], making it difficult to generate effective preventive measures to decrease the prevalence of its infection.

The stomach of humans is the natural habitat for *H. pylori*, which proliferates usually in the superior section of the intestine and in the gastric mucosa and causes the above-mentioned associated pathologies [11]. This pathogen possesses the metabolic machinery to grow in the gastric environment and to survive under the harsh conditions that exist there. The enzymatic arsenal that favors the growth of *H. pylori* in this anatomical site includes enzymes such as urease, carbonic anhydrases, catalase, peroxidase, and superoxide dismutase [11,18,19,20]. Since *H. pylori* adapts to the gastric conditions, extra-gastric conditions such as changes in pH and variations in oxygen concentration constitute stressors that impact its morphology and survival [21,22]. To survive in these environments, it can become part of a biofilm, develop a stage of viable but non-culturable bacteria (VBNC), or grow within eukaryotic microorganisms such as amoebas or yeasts [23,24,25,26,27], which could provide shelter from imminent threats to its viability.

Regarding the intracellular relationship of *H. pylori* and yeasts, it has been established that this bacterium can invade yeasts from different sources including the environment, foods, or the vaginal and oral microbiota of humans [28,29,30]. However, the environmental conditions that support the growth of *H. pylori* within these fungal cells still needs to be investigated in detail. Recently, it was reported that alterations in pH resulted in a stressful environment for *H. pylori* and increased its entry into yeast cells of the genus *Candida*, especially at low pH [31] values. Variations in the concentration of nutrients, temperatures outside the optimal range, as well as antibiotics such as amoxicillin have been shown to increase the growth of *H. pylori* within *Candida* cells [32,33,34]. There is limited knowledge about the relationship of intra-yeast *H. pylori* with the yeast cells that shelter it or how yeast cells might provide protection and serve as transmission vehicles for the bacterium, and therefore, its transmission routes should be investigated. It is also essential to determine the environmental factors involved in this pathogen harboring within yeast cells of the genus *Candida*. Thus, the aims of this work are: (i) to determine whether aerobic or anaerobic conditions are stressors which support the growth of *H. pylori* within *Candida* yeast cells, (ii) to identify if the endosymbiotic association between yeast and bacteria is dependent on the strain of both microorganisms, and (iii) to determine if *H. pylori* remains viable within yeast cells, sheltering it against unfavorable environmental oxygen variations.

## 2. Materials and Methods

### 2.1. Culture Conditions

In this work, the *H. pylori* strains used were the reference strains J99 (also referred as ATCC 700824), G-27 and SS-1, and the clinical strain H707 (gastric biopsy origin). In addition, two *Candida* reference strains (*Candida albicans* ATCC 90028 and *Candida glabrata* ATCC 90030) and two clinical strains of the same genus (*C. albicans* VT-3 (vaginal discharge origin) and *C. glabrata* LEO-37 (oral cavity origin)) were considered to be yeasts representative of the genus *Candida*. These strains are all maintained at the culture collection of the Laboratory of Bacterial Pathogenicity, Department of Microbiology, University of Concepcion, Chile.

The *H. pylori* strains were cultured on Columbia agar (CA) (OXOID, Basingstoke, UK) plus 5% FBS (Biological Industries, Cromwell, CT, USA) and the plates were incubated in a microaerobic incubator (10% CO_2_ and 5% O_2_) (Thermo Scientific, Waltham, MA, USA) at 37 °C from 48 h to 72 h [35]. Regarding the *Candida* strains, they were cultured in Sabouraud agar (SA) (Merck, Darmstadt, Germany) plus chloramphenicol (CHL) (OXOID, Basingstoke, UK), in accordance with the instructions of the manufacturer. This medium will be hereafter referred as SA-CHL. Plates were incubated in an aerobic incubator (ZHICHENG, Shanghai, China) at 37 °C for 24 h. To confirm the purity of the cultures of both microorganisms, the Gram staining was performed, and then observed using an optical microscope. Additionally, urease, oxidase, and catalase tests were performed to corroborate the purity of the cultures of the *H. pylori* strains. For the *Candida* strain cultures, the purity was verified culturing random yeast cells in CHROMagar (Difco, Wokingham, UK), conducting urease tests, and observing wet mounts, using the oil immersion objective lens of an optical microscope to confirm the absence of extracellular bacteria or of bacteria-like bodies (BLBs) contained in the vacuole of yeast cells [36].

### 2.2. Growth of H. pylori Strains and Candida Strains Cultured under Aerobic, Microaerobic, or Anaerobic Conditions

This assay was performed as described by Sánchez-Alonzo et al. [33] with modifications. Each strain of *H. pylori* and *Candida* strain was suspended at an optical density (O.D.) of 0.1 at 600 nm in Brucella broth (BB) (Difco, Wokingham, UK) plus 5% FBS (BB-5%FBS) and placed in an Infinite M200 PRO microplate reader (TECAN, Männedorf, Switzerland) at time zero. A 200 µL aliquot of each suspension was transferred to wells of flat bottom 96-well plates (Thomas Scientific, Swedesboro, NJ, USA). Different plates were used for each strain under three different conditions (aerobic, microaerobic, or anaerobic) which were incubated in the Infinite M200 PRO reader. The microaerobic and anaerobic conditions were generated using CampyGen and Anaerogen sachets (Thermo Scientific, Waltham, MA, USA), respectively. The microaerobic condition corresponded to 10% CO_2_ and 5% O_2_. To evaluate the growth of the strains, the absorbances of the cultures were measured at a wavelength of 600 nm in the same Infinite M200 PRO reader every 2 h for yeast strains and every 8 h for *H. pylori* strains. These measurements were made for 50 h and 72 h for the yeast and the bacterium, respectively. Growth curves for each strain were performed in triplicate. The methodology of this section is shown in Figure S1.

### 2.3. Co-Cultures of H. pylori Strains with Candida Strains

These assays were performed independently co-culturing each one of the *H. pylori* strains with each one of the different *Candida* strains, following the protocol described by Sánchez-Alonzo et al. [33]. Suspensions of each yeast and bacterial strain were adjusted to an O.D. of 0.1 at 600 nm in 0.89% saline solution (SS). Then, 500 µL of *H. pylori* strain suspension and 500 µL of *Candida* strain suspension were placed in the well of 12-well plates (Thomas Scientific, Swedesboro, NJ, USA) containing 4 mL BB-5%FBS. The plates were kept at 37 °C for 48 h under the aforementioned conditions. The microaerobic and anaerobic conditions were also achieved using CampyGen or Anaerogen sachets, respectively, as described in the section above. Each co-culture was performed in triplicate. The methodology of this section is shown in Figure S2.

### 2.4. Search for Intra-Yeast Bacteria-like Bodies (BLBs)

This assay was performed as described by Sánchez-Alonzo et al. [33] with modifications. At the same time of incubating the co-cultures, wet mounts of the co-cultures were prepared taking 20 µL aliquots from each co-culture at 0, 1, 3, 6, 12, 24, and 48 h. Each one of the aliquots was laid on a glass slide and observations were made using an optical photomicroscope (Leica, Wetzlar, Germany) using the 100X objective lens to search for mobile BLBs within yeast cells. If yeast cells harboring bacteria-like bodies (Y-BLBs) were observed, 20 µL of the respective co-culture were transferred to Sabouraud agar plus chloramphenicol (SA-CHL) and the cultures were incubated under aerobic conditions at 37 °C for 24 h. Once the incubation period was completed, wet mounts of yeast cells obtained from random colonies were observed to verify the presence of Y-BLBs in the cultures. Then, colonies from the same cultures were taken and placed in Eppendorf tubes containing 1 mL of 1X phosphate buffered saline (PBS) pH 7.4 plus 0.015 µL mL clarithromycin, and the tubes incubated at 37 °C for 24 h under microaerobic conditions (10% CO_2_, 5% O_2_). The yeast cells were washed using 1 mL of 1X PBS and centrifuging at 6700× *g* (Eppendorf, San Diego, CA, USA). An aliquot of 20 µL was obtained and streaked in SA-CHL containing plates which were incubated at 37 °C for 24 h. Once the yeast cells grew, wet mounts and Gram-staining of yeast cells were performed to verify the absence of extracellular bacteria. The methodology of this section is shown in Figure S3.

### 2.5. Identification of Intra-Yeast BLBs Using the FISH Technique

One milliliter of sterile 1X PBS was added to 2 mL Eppendorf tubes (Hauppauge, NY, USA), and then yeast cells were added from colonies selected at random from the cultures in SA-CHL in which co-cultures were positive for BLBs until their turbidity was similar to that of Tube 3 as the McFarland Standard. The tubes were centrifuged at 6700× *g* for 2 min, and this last step was repeated once. One mL of PBS was added to each one of the pellets and vortexed for 5 s (DLAB, Ontario, CA, USA). Then, 100 µL of each yeast cell suspension was placed on a glass slide, and allowed to dry for 20 min. Next, they were fixed and dehydrated as described by Böckelmann and coworkers [37], and dried at room temperature. One hundred µL of hybridization solution (270 µL 5 M NaCl, 30 µL 1 M TRIS-HCl, 525 µL of 37.7% deionized formamide, 675 µL of MiliQ water, and 1.5 µL of 10% SDS) and then 6 µL of 5 ng µL^−1^ Hpy probe 5′-CACACCTGACTGACTATCCCG-3′ labeled with Cy3 [38] were added to each smear. Hybridization and washing were also performed as described by the above-mentioned authors. After the last washing, the slides were allowed to dry and 100 µL of 1 mg mL^−1^ aniline blue was added, and then the slides were incubated at room temperature, for 10 min. The smears were washed two times using 1 mL of 1X PBS and they were allowed to dry in the darkness. The slides were observed using a camera-equipped fluorescence microscope (Motic, Viking Way, Richmond, BC, Canada) fitted with TRIC (AT540/605) and DAPI (AT395/460) filters (Motic, Viking Way, Richmond, BC, Canada). The images captured were processed and combined using the ImageJ software (NIH Image, Bethesda, MD, USA). The methodology of this section is shown in Figure S4.

### 2.6. Detection of the 16S rRNA Gene of H. pylori in the Total DNA of Yeast Cells

This assay was performed as described by Sánchez-Alonzo et al. [33] with modifications. Yeast cells from random colonies were taken from cultures on SA-CHL in which the presence of Y-BLBs was detected. Yeast cells were added to 1 mL of SS until an O.D. of 0.1 at 600 nm was obtained; each suspension was centrifuged at 6700× *g* for 2 min. Then, each pellet was resuspended in 1 mL of 10 mM Tris EDTA (TE) buffer adjusted to a pH of 8.0 and then agitated in a vortex for 5 s. Then, each solution was centrifuged at 11,300× *g* during 5 min, the supernatants discarded and 200 µL of TE buffer were added. Next, each tube was subjected to a heat shock which included freezing at −80 °C for 30 min and thawing in a thermoblock at 100 °C for 10 min; repeating this cycle thrice. After the third cycle, the tubes were incubated at −80 °C for 24 h, and then incubated at 70 °C during 2 h. Finally, the total yeast’s DNA was extracted using the commercial NucleoSpin Tissue kit (MACHEREY-NAGEL, Düren, Germany) following the manufacturer’s instructions. After the total DNA was extracted, the *16S rRNA* gene of *H. pylori* was amplified by PCR and the amplicons visualized in an agarose gel electrophoresis to detect the presence of the expected 110 pb amplicon, all this as described by Sánchez-Alonzo and coworkers [33], with few modifications: 1.5 μL of DNA samples were added, 30 amplification cycles were programmed and 5 mL of the amplified product were loaded in each lane. Finally, the amplicons were recorded exposing the gel to ultraviolet light in a ENDURO model photodocumenter (Labnet, Edison, NJ, USA). The design of the assays of this Section is shown in Figure S5.

### 2.7. H. pylori Viability Assay

This assay was performed as described by Sánchez-Alonzo et al. [33] with modifications. Yeast cells cultured in SA-CHL in which Y-BLBs were observed, a suspension with a turbidity similar to 0.5 MacFarland Standard was made in 1 mL of SS. Then, 1 µL of the working solution of a LIVE/DEAD BacLight Bacterial Viability Kit L-7012 (ThermoFisher, Waltham, MA, USA) was added. Suspensions were incubated for 15 min in the darkness, and agitated in a vortex at minimum speed for 3 s (DLAB, Ontario, CA, USA). Then, the pellets were resuspended, and 10 µL of each suspension were added to a glass slide which was placed under the 100x objective lens of a camera-equipped fluorescence microscope (Motic, Viking Way, Richmond, BC, Canada). The filters fitted to the microscope were FITC (AT480/535) and TRIC (AT540/605) filters. The images were processed and combined using the ImageJ software version 1.53 (NIH Image, Bethesda, MD, USA). The design of the assays of this section is shown in Figure S6.

### 2.8. Statistical Analysis

Data collected were analyzed using the SPSS 24.0 software (IBM Company, Armonk, NY, USA). The Tukey´s test was used to verify whether or not differences were significant. Values of *p* ≤ 0.05 were considered to be significant, while those ≤ 0.0001 were considered to be highly significant. Different letters in tables or figures indicate that, in accordance with the Tukey´s test, the results are significantly different.

## 3. Results

### 3.1. Growth Curves of H. pylori and Candida Strains Cultured under Aerobic, Microaerobic, or Anaerobic Conditions

**Figure 1:**
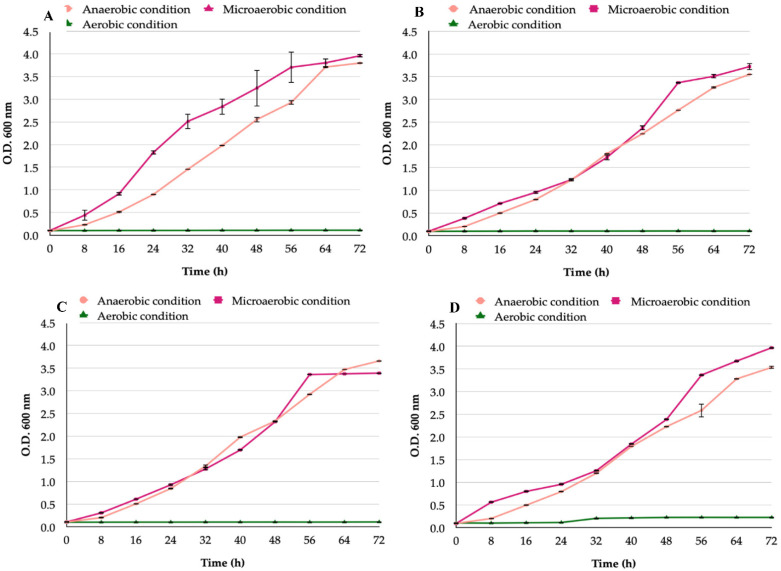
Growth curves of *H. pylori* strains incubated in the presence of an aerobic, microaerobic, or anaerobic condition: (**A**) *H. pylori* reference strain J99; (**B**) *H. pylori* reference strain G-27; (**C**) *H. pylori* SS-1 reference strain; (**D**) *H. pylori* H707 clinical strain.

There was no significant difference when comparing the in vitro growth curves of all *H. pylori* strains incubated under microaerobic or anaerobic condtions (Figure 1). The bacterial growth incubated under aerobic conditions was compared with that obtained under anaerobic or microaerobic conditions and highly significant inhibition (*p* < 0.0001) was observed (Figure 1). The growth curves of different *H. pylori* strains under the same incubation conditions were compared and there were no significant differences observed (*p* = 0.1).

**Figure 2:**
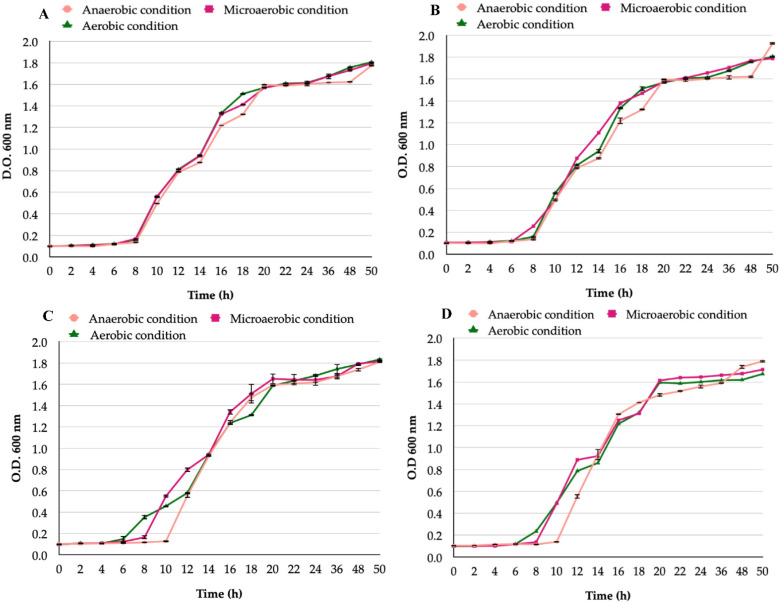
Growth curves of *Candida* strains under aerobic, microaerobic, or anaerobic conditions: (**A**) *C. albicans* ATCC 90028 reference strain; (**B**) *C. glabrata* ATCC 90030 reference strain; (**C**) *C. albicans* VT-3 clinical strain; (**D**) *C. glabrata* LEO-37 clinical strain. No significant differences were present among these growth curves (*p* = 0.5).

Regarding the growth of yeasts belonging to the *Candida* genus, all strains grew when incubated under the three conditions evaluated (Figure 2) with no significant differences in the growth obtained under each condition. The growth of all the *Candida* strains incubated in either of the three conditions tested showed no significant differences (*p* = 0.2).

### 3.2. Detection of Bacteria-like Bodies (Y-BLBs) within Yeasts

**Figure 3:**
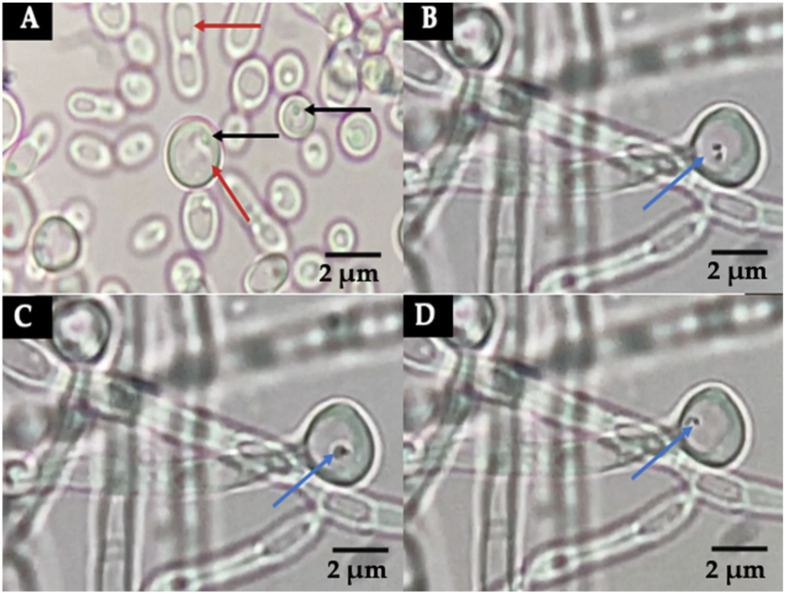
Wet mounts of yeast cells harboring bacteria-like bodies (Y-BLBs). Images, similar to observations of other co-cultures, correspond to a co-culture of *H. pylori* H707 strain and *C. albicans* VT-3 strain, incubated under an anaerobic condition: (**A**) Yeast cells from a pure culture of the *C. albicans* VT-3 cells lacking BLBs in their vacuole (red arrow), nuclei of the yeast cells indicated with black arrows; (**B**–**D**) show the changes in positions of BLBs in the vacuoles (blue arrows) in images taken at 1 s intervals. The actual movement of the BLBs is shown in Video S1.

**Figure 4:**
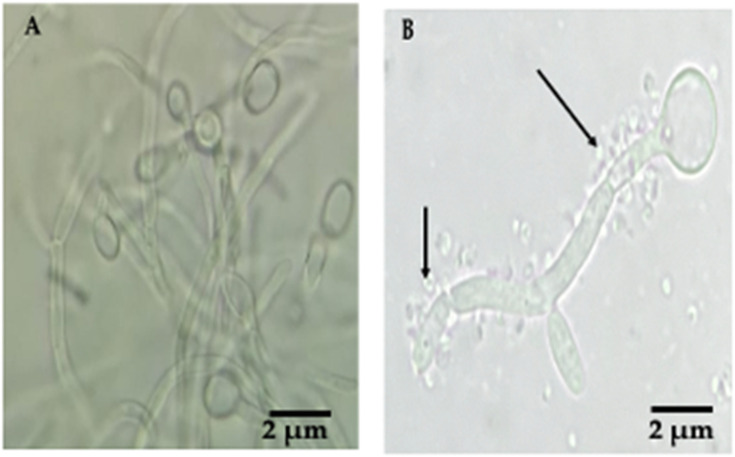
(**A**) Wet mount of a pure culture of *C. albicans* VT-3 cells, no extracellular bacteria nor bacteria adhered to the surface of hyphae can be seen; (**B**) wet mount of a co-culture of *H. pylori* G-27 strain and *C. albicans* VT-3 strain incubated under an anaerobic condition showing extracellular *H. pylori* bacteria adhered to the filamentous yeast structures (black arrow).

Mobile Y-BLBs were detected in the wet mounts of all co-cultures incubated under the three different conditions, starting at time 1 h. The movements of BLBs were ascertained observing the changes in positions of the BLBs within vacuoles of yeast cells (Figure 3B–D). Wet mounts also revealed *H. pylori* cells adhering to pseudohyphae of *C. albicans* cells (Figure 4).

**Figure 5:**
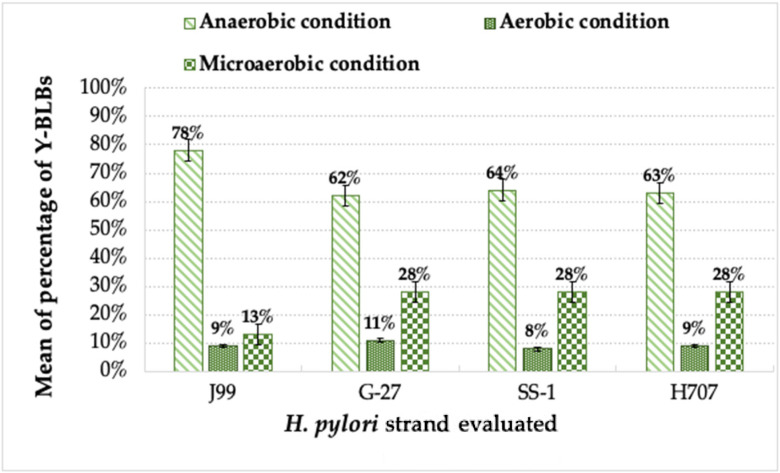
Means of the percentages of yeast cells harboring bacteria-like bodies (Y-BLBs) in co-cultures of *H. pylori* strains plus *Candida* strains incubated for 48 h under anaerobic, microaerobic, or aerobic conditions. The higher means of Y-BLB percentages were found in co-cultures including the *H. pylori* J99 strain incubated under anaerobic conditions.

When analyzing the percentages of Y-BLBs present in the different co-cultures, the total number of Y-BLBs present in the different incubation conditions, and the strain of *H. pylori* being evaluated, the higher Y-BLB percentages were obtained when incubation was conducted under anaerobic conditions (62% to 78%), followed by incubations under microaerobic (13% to 28%) or aerobic conditions (8% to 11%). The means of the Y-BLB percentages also varied according to the strain of *H. pylori* assayed. The higher means of the Y-BLB percentages were found in those co-cultures in which the *H. pylori* J99 strain was incubated under anaerobic conditions (Figure 5), being highly significantly different from the means of the Y-BLB percentages obtained when incubations were under aerobic or microaerobic conditions. The Y-BLB percentages found in co-cultures incubated under microaerobic conditions were, in all cases, significantly higher than those obtained when incubated under aerobic conditions (*p* = 0.03) (Figure 5).

**Figure 6:**
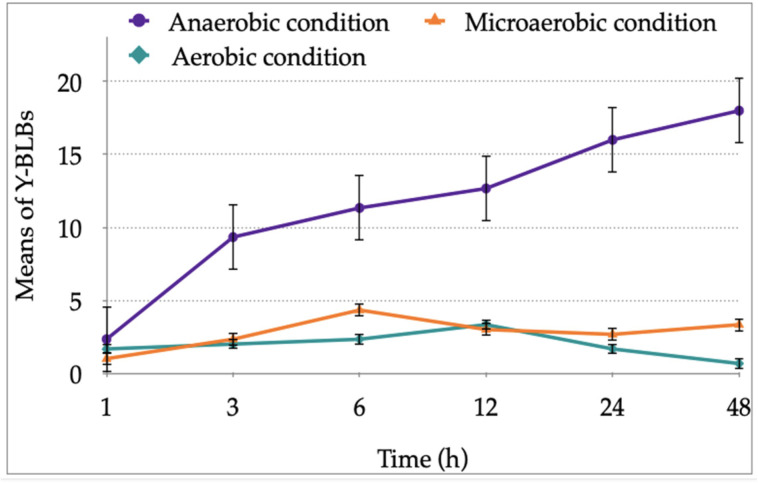
Means of yeast cells including bacteria-like bodies (Y-BLBs) found in the different co-cultures under the different incubation conditions. The higher means of Y-BLB percentages were reached at 24 h and 48 h of co-culture incubation under anaerobic conditions.

**Figure 7:**
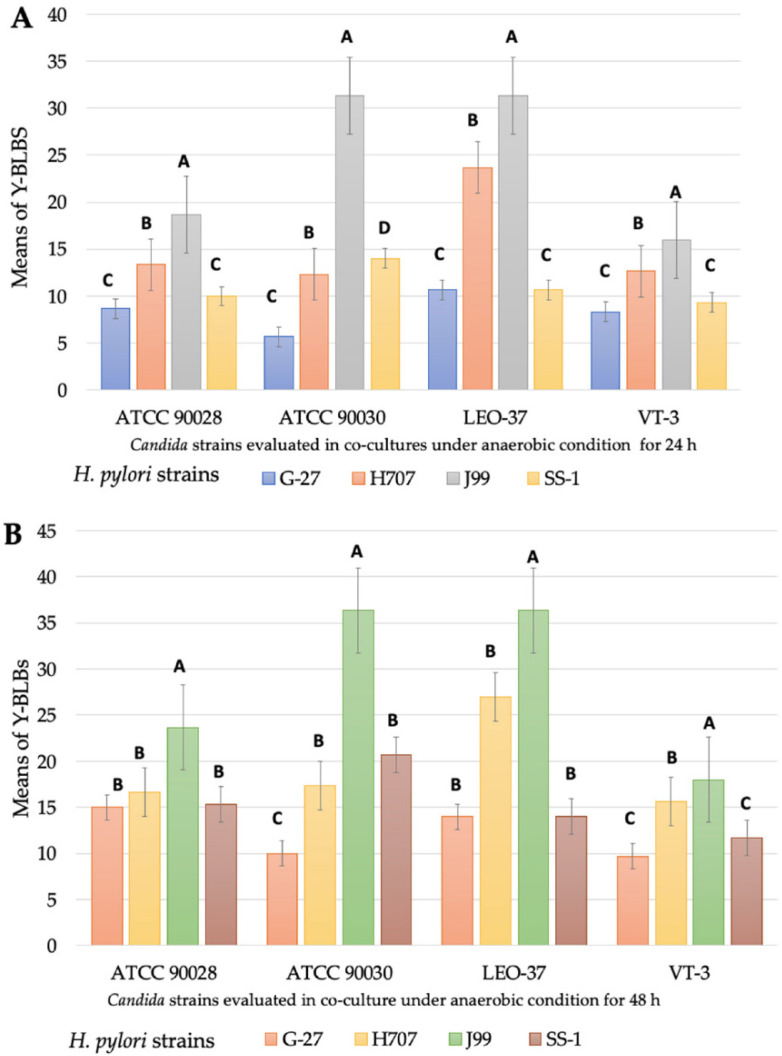
Means of intra-yeast bacteria-like bodies (Y-BLBs) found in *H. pylori* strains and *Candida* strains, co-cultured under anaerobic conditions for 24 h (**A**) or 48 h (**B**). At both incubation times, the higher means of Y-BLB percentages were found when the *H. pylori* J99 strain was co-cultured with *C. glabrata* strains. Different letters indicate significant differences (*p* < 0.05). Strains ATCC 90028 and VT-3 belong to *C. albicans* species and strains ATCC 90030 and LEO-37 belong to *C. glabrata* species.

When the means of the Y-BLB percentages obtained per co-incubation time were analyzed, it was observed that the higher means were obtained at 24 h and 48 h under anaerobic conditions (Figure 6). This information allowed us to establish that the *H. pylori*-*Candida* co-culture combination produced the higher means of Y-BLB percentages. The co-cultures combining the *C. glabrata* strain ATCC 90030 or the *C. glabrata* strain LEO-37 plus the *H. pylori* strain J99 resulted in the higher means of Y-BLB percentages (Figure 7).

### 3.3. Identification, at the Species Level, of Intra-Yeast Bacteria-like Bodies (BLBs)

**Figure 8:**
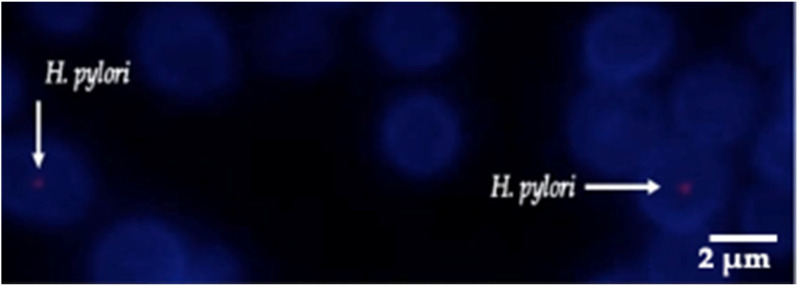
Yeast cells obtained from a *H. pylori* J99-*C. glabrata* LEO-37 co-culture incubated under anaerobic conditions in Brucella broth medium plus 5% fetal bovine serum. The red fluorescence observed within yeast cells is a consequence of the hybridization of the *H. pylori*-specific fluorescent probe (white arrow). Blue fluorescence corresponds to aniline blue bound to yeast 1,3 β-glucans.

**Figure 9:**
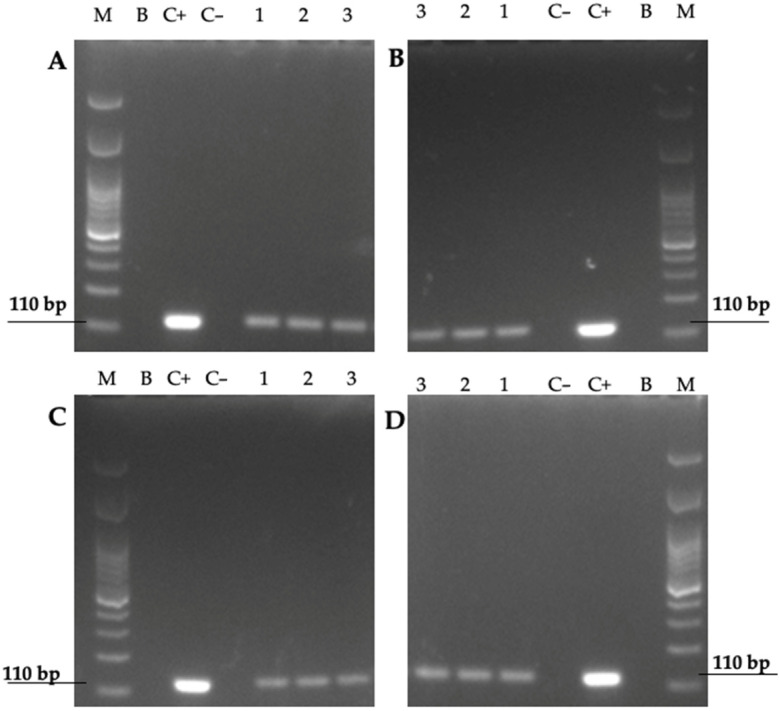
Images of 2% agarose gel showing the amplicons resulting from the amplification, by PCR, of the *16S rRNA* gene of *H. pylori* from the total DNA of different *Candida* strains previously co-cultured under different oxygen concentrations with *H. pylori* J99 strain: (**A**) *H. pylori* J99 strain and *C. albicans* ATCC 90028 strain co-culture; (**B**) *H. pylori* J99 strain and *C. glabrata* ATCC 90030 strain co-culture; (**C**) *H. pylori* J99 strain and *C. albicans* VT-3 strain co-culture; (**D**) *H. pylori* J99 strain and *C. glabrata* LEO-37 strain co-culture. M, markers of molecular weight; B, blank (master mix, primers, and PCR grade water); C−, negative control (pure *C. glabrata* LEO-37 strain DNA); C+, positive control (pure *H. pylori* J99 strain DNA). Lanes 1, 2 and 3: amplicons amplified from the total DNA extracted from yeast cells previously co-cultured with *H. pylori* under aerobic, microaerobic, or anaerobic conditions, respectively.

The fluorescent in situ hybridization (FISH) technique revealed that yeast cells previously found to contain BLBs showed the expected red fluorescence corresponding to the Cy3-labeled fluorescent probe specific for *H. pylori* DNA. This assay allowed us to identify BLBs as cells belonging to the species *H. pylori* growing inside the *Candida* cells (Figure 8). Furthermore, the identification, at the species level, of Y-BLBs as *H. pylori* was also supported by the amplification of the *H. pylori* gene codifying its *16S rRNA* in the total DNA extracted from yeast cells which were previously co-incubated with *H. pylori* cells, and then the presence of Y-BLBs was confirmed (Figure 9). Together, both assays confirmed that the BLBs observed under an optical microscope corresponded to intracellular *H. pylori* within *Candida* cells.

### 3.4. Viability Assessment of H. pylori Cells Harboring in Yeast Cells

**Figure 10:**
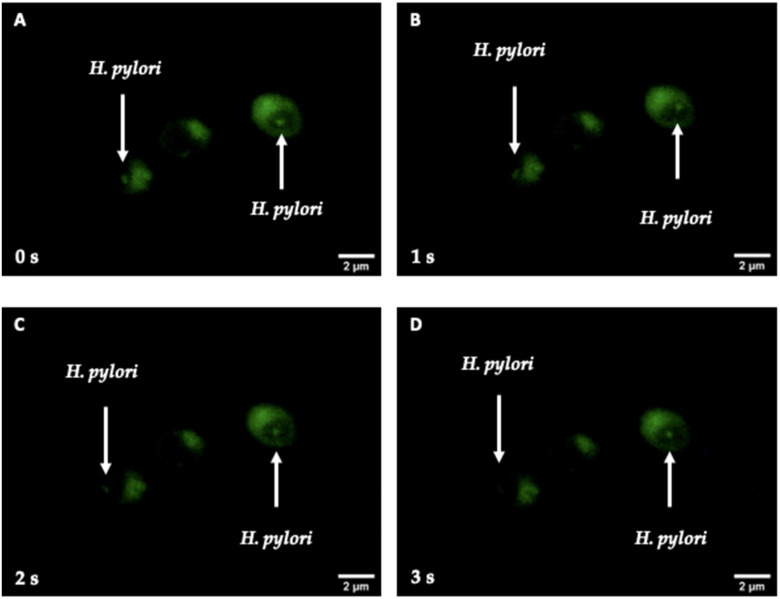
Fluorescence microscopy of *C. glabrata* LEO-37 strain cells co-cultured with *H. pylori* G-27 strain cells under microaerobic condition for 48 h, and then cultured on Sabouraud agar plus chloramphenicol for 24 h to eradicate bacteria extracellularly located. The viability of bacteria harboring within *Candida* cells was demonstrated by SYTO 9 green fluorescence within yeast cells (white arrows). Images (**A**–**D**) were obtained from the same microscopic field at 1 s intervals showing the change of position of bacteria located in the vacuole of *Candida* cells.

The LIVE/DEAD BacLight Bacterial Viability Kit demonstrated, by the SYTO 9 green fluorescence present in the interior of the vacuoles of yeast cells, that viable *H. pylori* cells harbor within yeast cells. Thus, intra-yeast *H. pylori* (or BLBs) remained viable after their entry into yeast cells and reseeding of the Y-BLBs in Sabouraud plus chloramphenicol (SA-CHL) (Figure 10). Furthermore, this same figure shows, at 1 s intervals, the change of position of the green fluorescent bodies (*H. pylori* cells) contained by the vacuoles of yeast cells, which indicated that bacteria were mobile.

## 4. Discussion

*H. pylori* is a pathogen that is well adapted to survive gastric conditions [22,39], and is difficult to grow in culture [40,41,42,43]. In vitro, this fastidious pathogen grows under microaerophilic and capnophilic conditions [44]. *H. pylori* growth under an aerobic condition showed a highly significant inhibition (*p* < 0.0001) as compared with the growth obtained when it was incubated under microaerobic or anaerobic conditions. Since *H. pylori* cells were able to survive under anaerobic conditions for up to 72 h, our results are in agreement with the literature [45]. It was not surprising that none of the *H. pylori* strains grew when incubated under aerobic conditions. Regarding *Candida* strains, all strains grew under the three incubation conditions studied. Our results are supported by studies in the literature reporting that although yeasts belonging to this fungal genus are classified as aerophilic, they have the ability to adapt to different environmental conditions, such as different oxygen concentrations [46,47,48]. Thus, *Candida* yeasts can be members of the normal human microbiota of the skin, mouth, vagina, and gastrointestinal tract [46,47,49].

*H. pylori* and *Candida* co-cultures incubated under anaerobic conditions showed that *H. pylori* J99 produced higher means of Y-BLB percentages regardless of the *Candida* strain used in the co-culture. Although, currently, there is no explanation for this, it has been reported that the invasiveness of the *H. pylori* J99 strain is higher than that of the G-27 and SS-1 strains [50]. Different bacteria synthesize chitinase which is used to degrade chitin to obtain carbon and nitrogen, and has also been reported to be a virulence factor which allows *Legionella pneumophila* to move across the alveolar mucosa [51,52]. It would be interesting to investigate if *H. pylori* strains produce chitinase to degrade chitin, a component of the yeast wall, favoring its entry into yeast cells.

Yeast factors could also be implicated in the *H. pylori* and *Candida* cell interactions. In the present work, the higher means of Y-BLB percentages were obtained when *H. pylori* J99 cells were co-cultured with the *C. glabrata* strains (LEO-37 or ATCC 90030). In 1998, Ansorg and coworkers reported [53] an *H. pylori* relationship with *C. glabrata*, describing the preference of bacterial adhesion towards non-*albicans Candida* yeasts. To date, the reason for the affinity between *H. pylori* and *C. glabrata* has not been clarified; however, it is possible to attribute it to the high production of adhesins by *C. glabrata* cells, which might provide a wide range of possibilities for bacteria to adhere to yeast. Furthermore, *C. glabrata* cells may have receptors on their surfaces that detect microbe-associated molecular patterns (MAMPs), as reported for the fungus *Fusarium graminearum* [54], which may lead *Candida* cells, after recognizing *H. pylori* MAMPs, to trigger a signaling cascade triggering bacterial endocytosis by the fungal cell; however, so far this is just a hypothesis. It is known that when subjected to varying environmental conditions, such as hypoxic conditions, yeast cells, including those of *C. glabrata*, express moonlighting proteins on their cell surfaces that participate in both adhesions and interactions with other cells and in the formation of a biofilm [55], which would favor interactions between bacteria and this species of yeast. The presence of hyphae in *C. albicans* favors bacterial adhesion to this yeast species and contributes to disseminate mobile bacteria [56], such as *H. pylori*. Furthermore, it has been described that hyphae could be a rich source of nutrients for bacteria [56].

In the present study, we report that higher means of Y-BLB percentages were obtained when co-culturing bacterial and yeast cells under anaerobic conditions. When oxygen in the environment is less than 1%, *H. pylori* morphologically transforms to a coccoid form [45]. However, as mentioned above, we achieved anaerobic conditions using AnaeroGen sachets, which generated a concentration from 7% to 15% CO_2_ and less than 0.1% oxygen. This may favor the growth of *H. pylori* outside yeast cells; however, the lack of the oxygen concentration required by this bacterium may be stressful for it, promoting its entry into yeast cells.

The second incubation condition evaluated was microaerobic condition, where a reduction in the means of Y-BLB percentages was observed as compared with those obtained under anaerobic conditions. These results are in agreement with the literature, since a microaerobic condition is considered to be the optimal environment for the growth of this pathogen, and therefore, *H. pylori* bacteria enters yeasts of the genus *Candida* without any apparent stress factor forcing the bacteria to search for shelter. These results are similar to those obtained in a previous study by our research group [31], where it was observed that under optimal pH growth conditions for *H. pylori* (pH 7), this bacterium enters into yeast cells, but at a lower rate as compared with conditions that threaten bacterial viability, such as acidic pH (pH 3 or 4). Similar results were observed under conditions of variations in nutrient concentrations [33].

The third incubation condition assayed was the aerobic condition, where percentages from only 8% to 11% Y-BLBs were obtained, percentages even lower than those recorded under anaerobic conditions. *H. pylori* is a bacterium which is sensitive to oxygen concentrations (from 20% to 21%) [57] that promote a coccoid form, VBNC condition, and also inhibit bacterial growth, clearly stressing this pathogen. Although no growth of *H. pylori* was observed under this environmental condition, there are studies in the literature that support its viability in the presence of oxygen from 6 h to 12 h. The above, together with the results of the present study, allow us to suggest that viable *H. pylori* cells could have entered into the yeast cells during the first hours of incubation of the co-cultures. This suggestion is supported by our observations of wet mounts prepared during the initial hours of co-cultures incubated under aerobic conditions that showed extracellular *H. pylori* cells with a bacillar morphology.

The identification, at the species level, of the BLBs using the FISH and PCR techniques together with the cell viability assay, allowed us to confirm that the intra-yeast BLBs observed in the wet mounts corresponded, in fact, to *H. pylori* viable cells with the ability to remain viable within this eukaryotic microorganism. This observation provides the basis for future research on the mechanism involved in the endosymbiosis between these two microorganisms, and to elucidate whether this mechanism is, in fact, another route of transmission for *H. pylori*.

## 5. Conclusions

*H. pylori* enters yeast cells of the genus *Candida* in a basal amount even if nonapparent stress conditions are present; however, anaerobic conditions significantly promote the entry of this bacterium into yeast cells. Moreover, under an anaerobic environment, the endosymbiotic relationship between *H. pylori* and *Candida* cells is *H. pylori* strain dependent, i.e., *C. glabrata* is preferred over *C. albicans* by *H. pylori*, particularly by *H. pylori* J99. Moreover, *H. pylori* is not only able to enter into *Candida* cells, mostly when subjected to a stress, but also remains viable within yeast cells, which could be used as a shelter.

## Data Availability

Not applicable.

## Supplementary Materials

The following supporting information can be downloaded at https://www.mdpi.com/article/10.3390/biology11050738/s1, Video S1: Wet mount of yeast cells harboring bacteria-like bodies (Y-BLBs) observed in a *H. pylori* H707-*C. albicans* VT-3 co-culture incubated under an anaerobic condition https://youtu.be/5iRM_Clkk4Y (accessed on 9 April 2022), Figure S1: Schematic representation of the methodology to obtain the growth curves of *H. pylori* and *Candida* strains cultured under aerobic, microaerobic, or anaerobic conditions, Figure S2: Schematic representation of the methodology to co-culture *H. pylori* strains with *Candida* strains, Figure S3: Schematic representation of the methodology to search for intra-yeast bacteria-like bodies (BLBs), Figure S4: Schematic representation of the methodology to identify the intra-yeast bacteria-like bodies (BLBs) using the FISH technique, Figure S5: Schematic representation of the methodology to detect the 16S rRNA gene of *H. pylori* in the total DNA of yeast cells, Figure S6: Schematic representation of the methodology to confirm the viability of intra-vacuolar *H. pylori* within yeast cells.
