# Supplementary material for: An Anaerobic Environment Drives the Harboring of Helicobacter pylori within Candida Yeast Cells
Source: Biology (Basel). 2022 May 12;11(5):738. doi: 10.3390/biology11050738 (PMC9139145; doi:10.3390/biology11050738)
Supplement: Supplementary file 1 [file biology-11-00738-s001.zip › biology-1709860-supplementary.pdf]

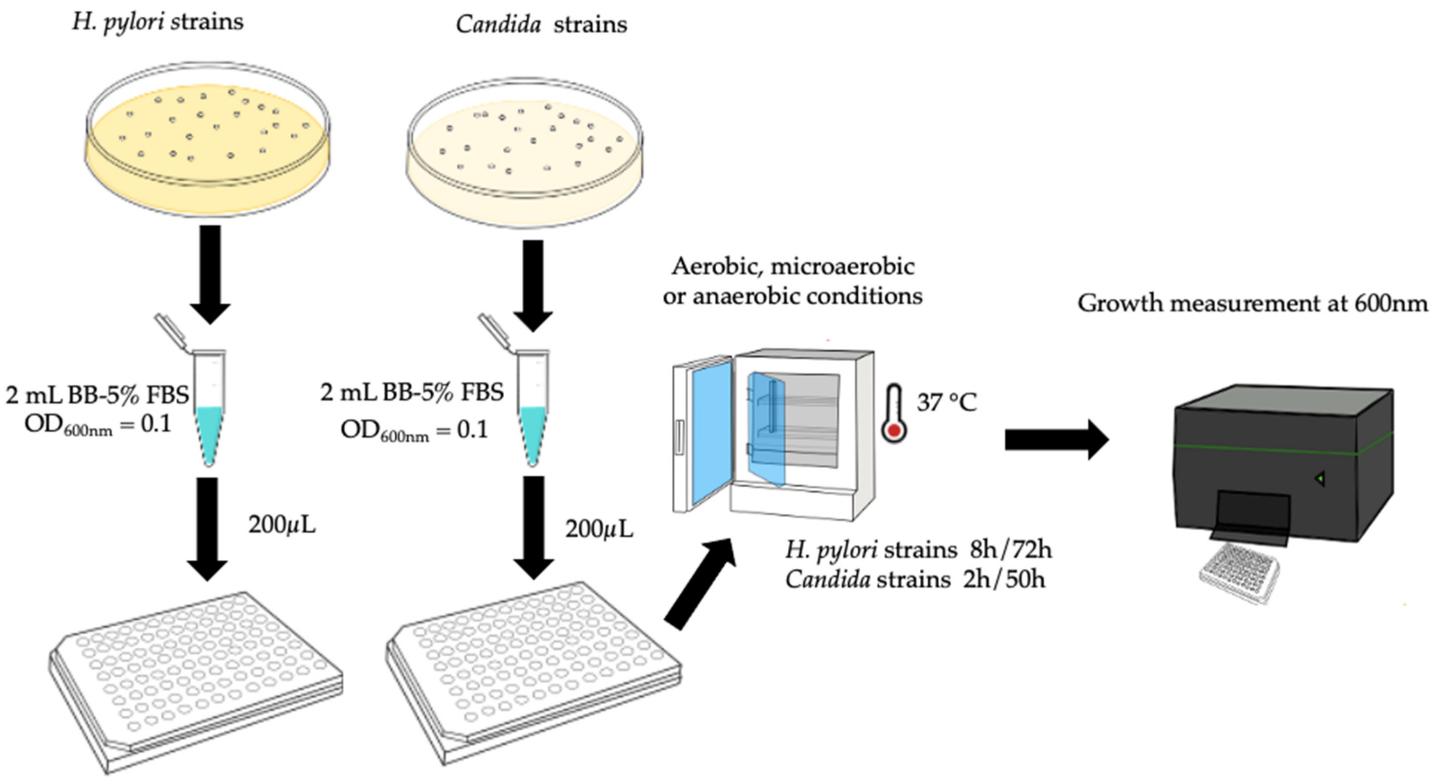

Figure S1. Schematic representation of the methodology to obtain the growth curves of *H. pylori* and *Candida* strains cultured under aerobic, microaerobic or anaerobic conditions.

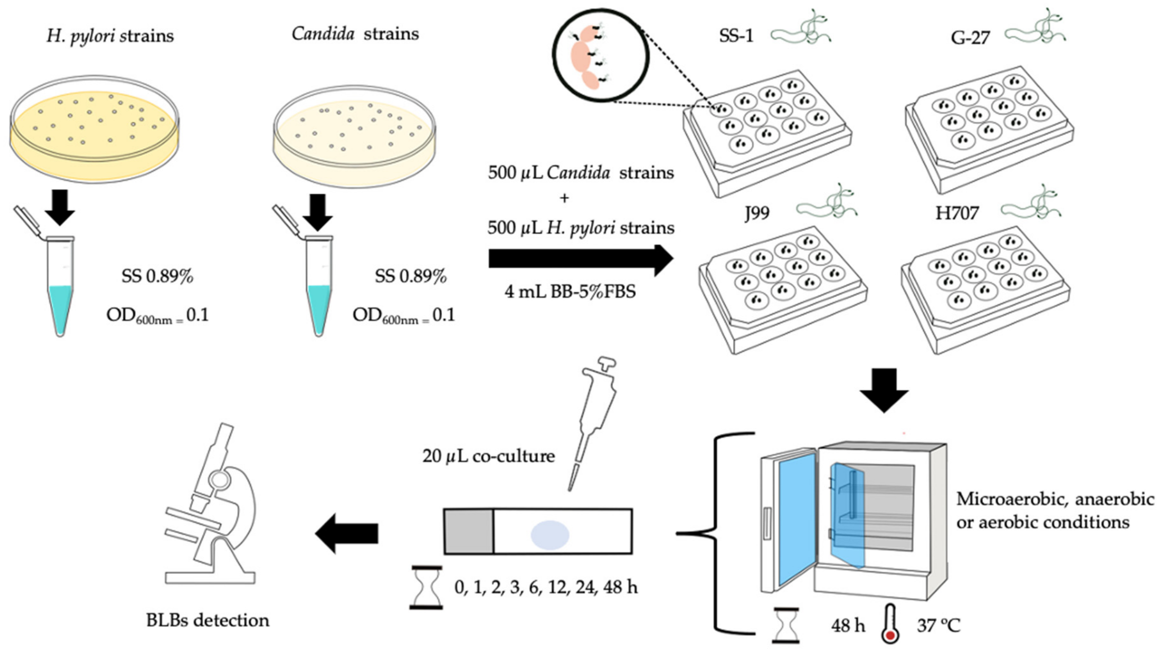

Figure S2. Schematic representation of the methodology to co-culture *H. pylori* strains with *Candida* strains.

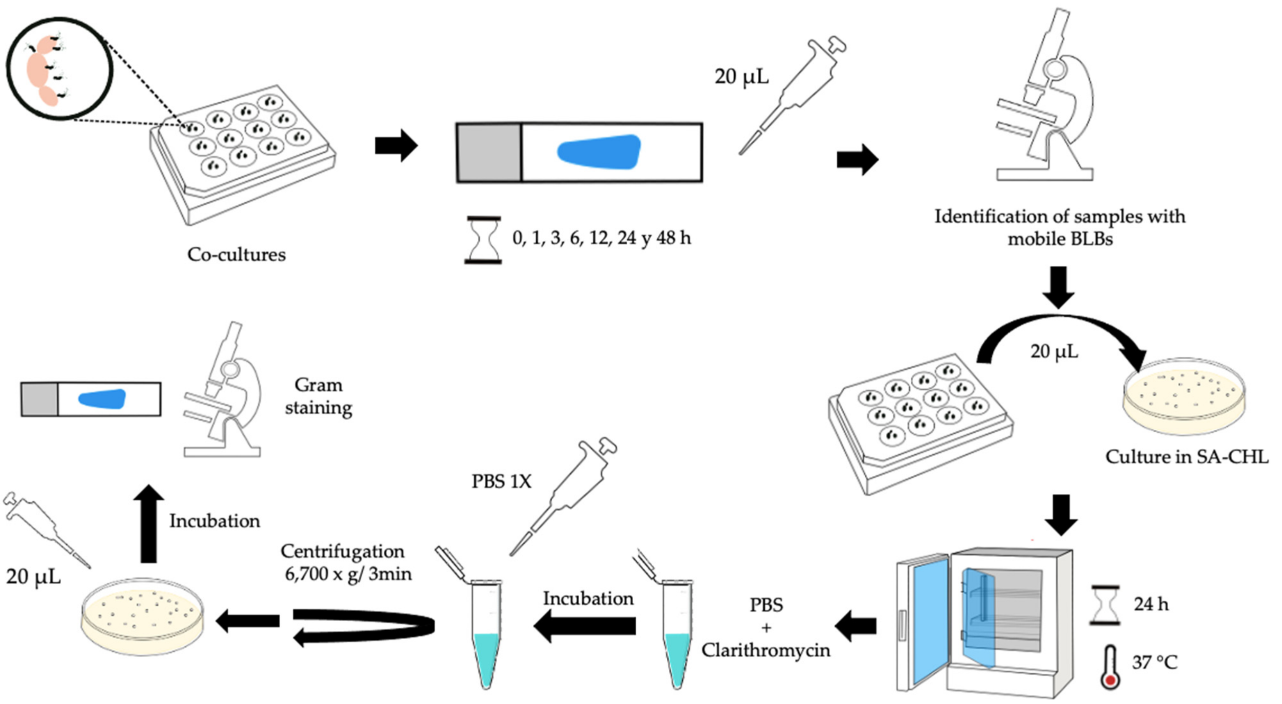

Figure S3. Schematic representation of the methodology to search for intra-yeast bacteria-like bodies (BLBs).

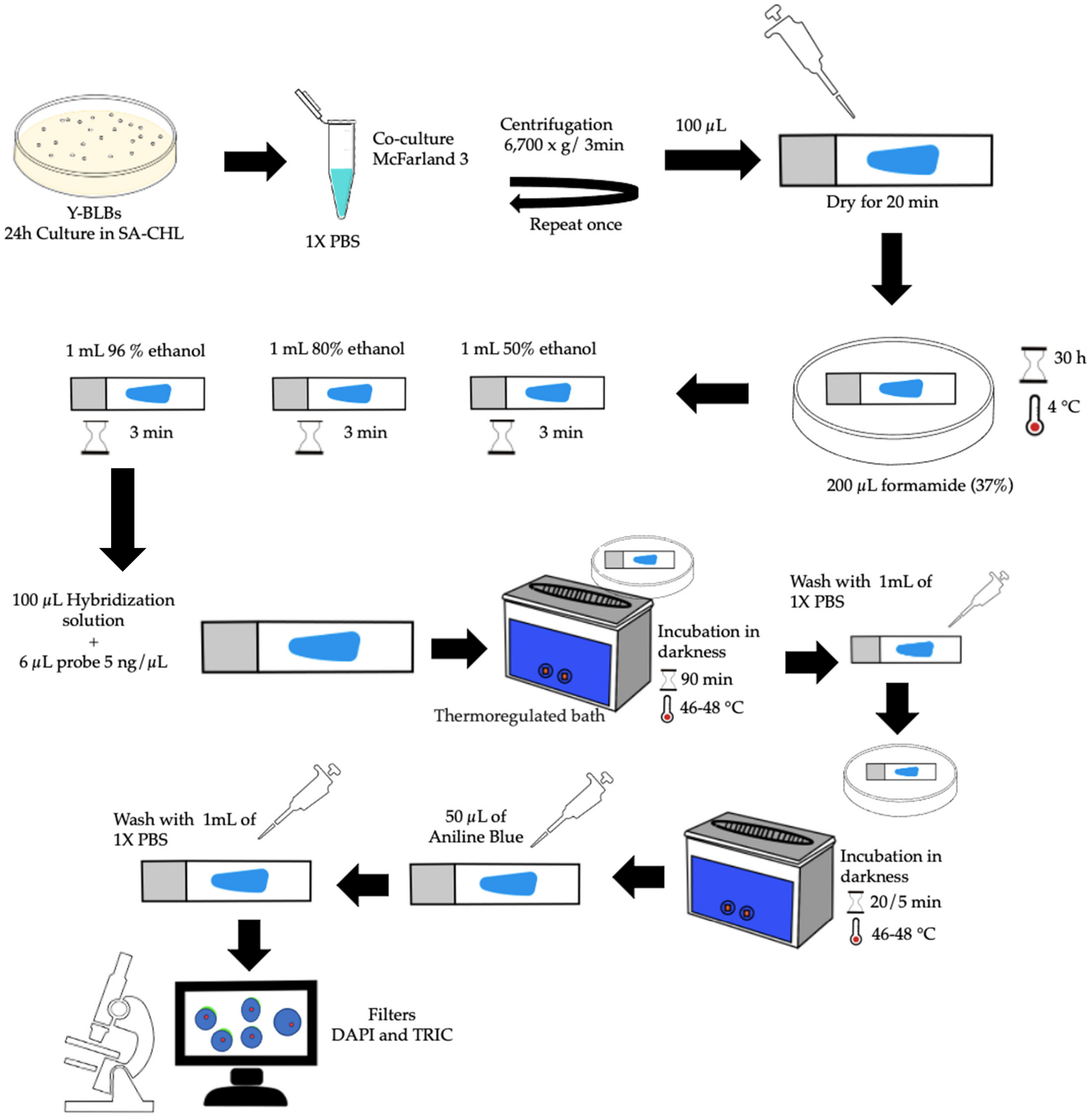

Figure S4. Schematic representation of the methodology to identify the intra-yeast bacteria-like bodies (BLBs) using the FISH technique.

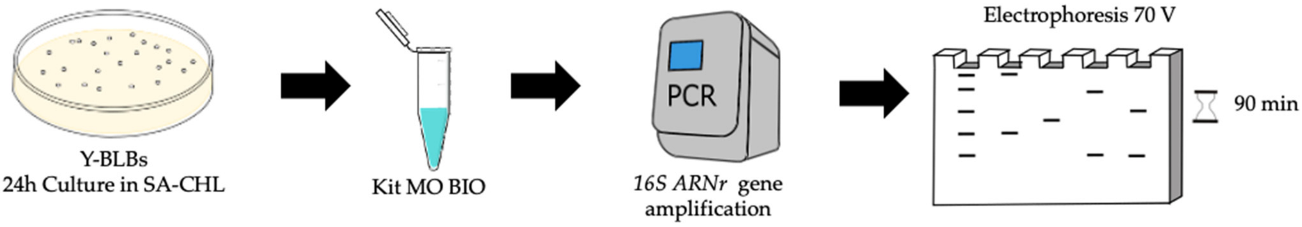

Figure S5. Schematic representation of the methodology to detect the *16S rRNA* gene of *H. pylori* in the total DNA of yeasts cells.

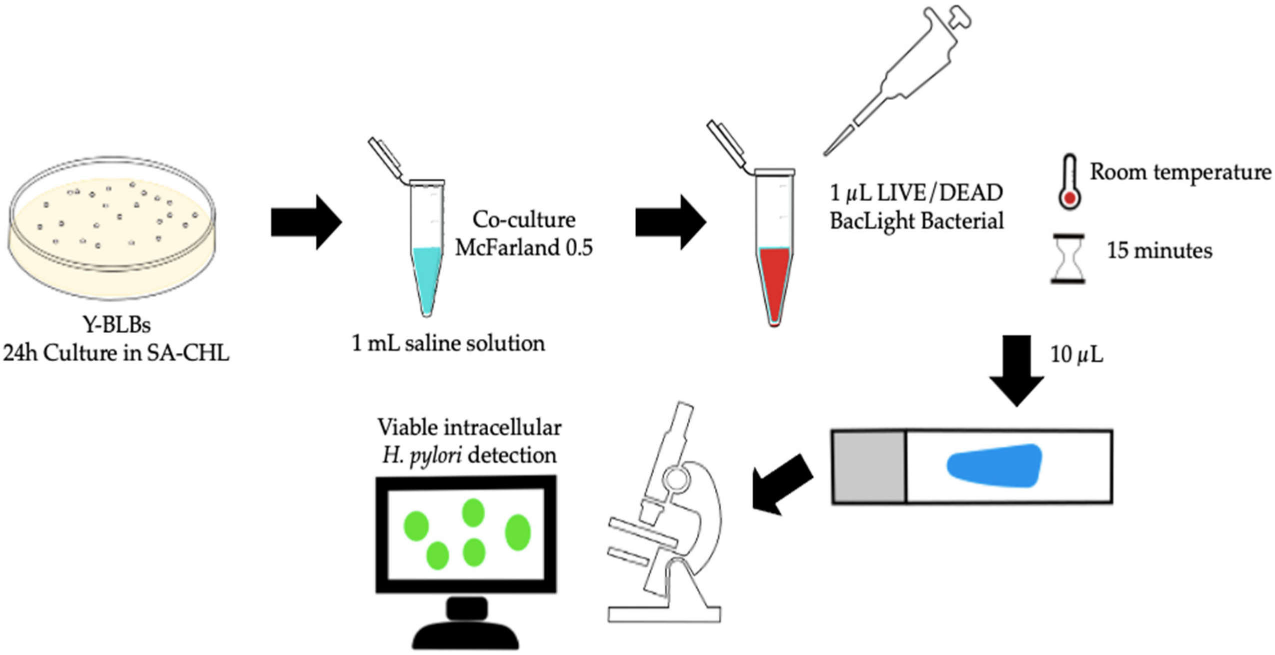

Figure S6. Schematic representation of the methodology to confirm the viability of intra-vacuolar *H. pylori* within yeasts.
